# Analysis of the Bacterial Flora of Substrates Used for the Cultivation of *Agaricus bisporus*: Relationship between *Clostridia* and Yield

**DOI:** 10.1264/jsme2.ME22041

**Published:** 2023-07-15

**Authors:** Daishin Tomikawa, Hiroshi Okuda

**Affiliations:** 1 Department of Bioscience and Engineering, Graduate School of Engineering and Science, Shibaura Institute of Technology, 307 Fukasaku, Saitama-shi, Saitama 337–8570, Japan

**Keywords:** *Agaricus bisporus*, bacterial flora, *Clostridium*, *Lactobacillus*, 16S rRNA gene ana­lysis

## Abstract

*Agaricus bisporus* has a high nutritional value and health benefits and its popularity is increasing among vegans and health-conscious consumers, indicating the need for its stable production. Therefore, we examined the bacterial flora of the substrates used to produce *A. bisporus* using a 16S rRNA gene ana­lysis and discussed the relationship between the bacterial flora and yield. The results obtained showed that *A. bisporus* yield slightly decreased with an increase in the abundance of *Clostridia* in substrates after primary fermentation. *Lactobacillus* showed little or no relationship with *A. bisporus* yield. *Clostridia* was identified as an indicator of *A. bisporus* yield.

*Agaricus bisporus* is one of the most widely cultivated mushrooms in the world (Ohga *et al.*, 1998). Although there are more than 3,000 edible mushroom species, *A. bisporus*, which has a high nutritional value and health benefits, is becoming increasingly popular among vegans and health-conscious consumers and may dominate the global market by 2025 (Sassine, 2021). It is also regarded as a meat substitute with similar nutritional value to several vegetables (Chang and Miles, 2004). Therefore, the stable production of *A. bisporus* is required to meet future demands.

Previous studies reported that substrates prepared from fermented horse manure for the cultivation of *A. bisporus* were of low quality due to poor fermentation, which negatively affected its yield (Sharma, 1991; Zhang *et al.*, 2014; Sassine, 2021). Therefore, the underlying reasons need to be examined.

A similar study reported the poor fermentation of silage. Silage is a livestock feed prepared from forage crops or grasses and produced by microbial fermentation (Yimi, 2002). The poor fermentation of silage is caused by bacteria belonging to the genus *Clostridium* (Yimi, 2002; Inoue *et al.*, 2012). *Lactobacillus* has been used to prevent the poor fermentation of silage (Kumai *et al.*, 1990). Although silage and the substrates of *A. bisporus* cultivation differ in their intended applications and materials used, both share the need for microbial fermentation. Based on this background, we hypothesized that the mechanisms responsible for the poor fermentation of substrates used in the cultivation of *A. bisporus* may be similar to those of silage. However, the impact of *Clostridium* and *Lactobacillus* on *A. bisporus* cultivation has not yet been examined in detail.

Substrates used for *A. bisporus* cultivation include horse manure, chicken manure, and cow manure. *A. bisporus* has traditionally been grown on a mixed substrate of horse manure and wheat straw (Farnet *et al.*, 2013), and the present study focused on horse manure. The preparation of this substrate is divided into primary and secondary fermentation phases. During primary fermentation, heat is generated by the activity of thermophilic bacteria, and the temperature of the growing substrate increases to >60°C (Sassine, 2021). Microorganisms break down carbohydrates and proteins, enabling the availability of nutrients in the substrate to *A. bisporus* (Ekman, 2017). During secondary fermentation, microorganisms break down refractory organic matter, such as cellulose and lignin (Zhang *et al.*, 2014; Kertesz and Thai, 2018; Cao *et al.*, 2019). Secondary fermentation occurs indoors and the substrate is not stirred. The substrate is then aged at 48°C to kill pathogens and pests and remove free ammonia (Omori and Koide, 2019; Suwannarach *et al.*, 2022). Based on these findings, the reasons for the poor fermentation of substrates used for *A. bisporus* cultivation may be similar to those of silage; however, the stage of poor fermentation that adversely affects the cultivation and yield of *A. bisporus* remains unclear. Therefore, the present study examined the bacterial flora of primary and secondary post-fermentation substrates and investigated the relationships between the relative abundance of *Clostridium*, *Lactobacillus*, and other bacteria involved in cultivation of *A. bisporus* and its yield.

The substrates used for *A. bisporus* production that underwent primary and secondary fermentation were supplied by *A. bisporus* farmers A, B, and C in Chiba Prefecture, Japan. Farmers A and B have high, stable yields, whereas farmer C has low, unstable yields. All farmers used horse manure, rice straw, and also the same processes in primary and secondary fermentation. In primary fermentation, farmers added water while stirring the substrate used for *A. bisporus* production until its color changed from brown to black. After the completion of primary fermentation, each sample was collected from three locations of the substrate (farmer A: *n*=3, farmer B: *n*=3, farmer C: *n*=3). In secondary fermentation, farmers packed the substrate used for *A. bisporus* production into a shelf in-house. After aging the substrate, each sample was collected from three locations on one shelf covered with the substrate (farmer A: *n*=3, farmer B: *n*=3, farmer C: *n*=3). All samples were collected from approximately 5‍ ‍cm inside the substrate surface around June in 2021 and immediately frozen. Each sample weight was approximately 30 g. The yields of farmers A, B, and C were the total amount of *A. bisporus* harvested from the substrates. Yield was expressed as the total *A. bisporus* weight per cultivated area. After thawing frozen samples and removing rice straw that remained in shape, 0.2‍ ‍g of the substrate was used for DNA extraction. DNA was extracted from each sample using the NucleoSpin®DNA Stool Kit, and the gene amplicon sequencing of extracted DNA was contracted to Genome-Lead Inc. A composite pair of primers comprising unique 17- or 21-base adapters was used to amplify the V3–4 region of the bacterial 16S rRNA gene from each sample. The forward primer sequence was 5′-TCGTCGGCAGCGTCAGATGTGTATAAGAGACAGN*CCTACGGGNGGCWGCAG*-3′, with the italicized sequence corresponding to the global broadly conserved bacterial primer F341. The reverse primer sequence was 5′-GTCTCGTGGGCTCGGAGATGTGTATAAGAGACAGN*GACTACHVGGGTATCTAATCC*-3′, with the italicized sequence representing the universal broadly conserved bacterial primer 806R. A second PCR amplification was performed using primers containing adapter and index sequences. Gene amplicon sequencing was performed under a 2×301-bp paired-end run on the Miseq system (Illumina). The MiSeq sequencer was used to sequence a library of the bacterial 16S rRNA gene V3–4 region. The resulting sequences were then analyzed using QIIME 2.

The relative abundance of bacteria is represented in the figure as the mean±standard error. An ana­lysis of variance with the Kruskal–Wallis test was performed to compare the mean relative abundance of bacteria among substrate samples collected from the farmers. Statistical ana­lyses were conducted using SPSS version 28.0.1.1 (14).

We compared the relative abundance of the bacterial phyla present in the substrates used to produce *A. bisporus* after primary and secondary fermentation (Fig. 1A and B). The relative abundance of *Firmicutes* was high in the substrates from all farmers after primary fermentation, but decreased to less than 10% after secondary fermentation. The bacterial flora at the phylum level of the primary and secondary post-fermentation substrates did not significantly differ among the farmers.

We compared the mean relative abundance of bacterial classes or orders present in the substrates used to produce *A. bisporus* after primary fermentation (Fig. 2A and Table 1). The average relative abundance of *Clostridia*, the bacteria responsible for the poor fermentation of silage, was high in the substrate from farmer C. We performed the Kruskal–Wallis test using the relative abundance of *Clostridia* present after primary fermentation in the substrates from farmers A, B, and C, which revealed a near-significant trend‍ ‍(*p* = 0.076) between farmers A and C. *Bacilli* and *Actinobacteria* are substrates used to produce *A. bisporus* (Yamauchi *et al.*, 2017; Omori and Koide, 2019; Sassine, 2021). In the present study, the mean relative abundance of *Bacilli* was >14% in all substrates, while that of *Actinobacteria* was <3%. We considered the stirring of substrates during primary fermentation to increase the abundance of aerobic bacteria, such as *Bacilli*. The mean relative abundance of *Lactobacillales*, which inhibit the growth of *Clostridia* in silage (Kumai *et al.*, 1990), was <1% in all substrates. *Pseudomonadales* are pathogens that cause brown blotch disease in *A. bisporus* (Miyazaki, 2018; Kuroda *et al.*, 2019), and the mean relative abundance of *Pseudomonadales* in the present study was <4% in all substrates.

We then compared the mean relative abundance of bacterial classes or orders present after secondary fermentation in the substrates (Fig. 2B and Table 1). The mean relative abundance of *Clostridia* in all substrates was <0.3%, which was significantly different from that present after primary fermentation. The mean relative abundance of *Bacilli* was lower and that of *Actinobacteria* was higher after secondary fermentation than after primary fermentation. Although the lack of stirring during secondary fermentation may have affected the abundance of aerobic bacteria in the substrates tested, these species remained predominant. The mean relative abundance of *Lactobacillales* was <0.02%, while that of *Pseudomonadales* was <0.7% in all the substrates. The mean relative abundance of *Lactobacillales* in the primary and secondary post-fermentation substrates was low‍ ‍and similar for all farmers. Secondary fermentation, including sterilization, markedly reduced the mean relative abundance of pathogens, such as *Pseudomonadales*. This may also account for *Lactobacillales* and *Pseudomonadales* showing little or no relationship with the yield of *A. bisporus*. The relationship between the abundance of *Clostridia* in the substrates after fermentation and the yields of *A. bisporus* are shown in Fig. 3. These yields were obtained from cultivation with the substrates used for the ana­lysis of the bacterial flora. The higher mean relative abundance of *Clostridia* present in the substrates after primary fermentation slightly decreased the yield of *A. bisporus*, suggesting a relationship between the yield of *A. bisporus* and the relative abundance of *Clostridia* after primary fermentation. Therefore, even if numerous *Bacilli* and *Actinobacteria* exist, which are useful for cultivation with the substrates, the yield of *A. bisporus* may decrease if there is an excess of *Clostridia* in the substrate after primary fermentation. The relative abundance of *Clostridia* in the substrate after second fermentation was low and similar in all farmers, whereas yields differed. Therefore, the yield of *A. bisporus* did not appear to correlate with the relative abundance of *Clostridia* in the substrates after secondary fermentation.

We examined the bacterial flora of the substrates used for *A. bisporus* cultivation and found a relationship between the abundance of *Clostridia* and the yield of *A. bisporus*. The yield of *A. bisporus* slightly decreased with increases in the abundance of *Clostridia* present in the substrates after primary fermentation. *Lactobacillus*, which inhibits the growth of *Clostridia* in the fermentation of silage, was virtually absent in the substrates. A relationship was not observed between the abundance of *Bacilli* or *Actinobacteria* present in the substrates and the yield of *A. bisporus*. Collectively, the present results may be useful for improving the productivity of *A. bisporus* using fermented substrates. All novel sequence raw data have been deposited in DDBJ (accession number DRA016706)

## Citation

Tomikawa, D., and Okuda, H. (2023) Analysis of the Bacterial Flora of Substrates Used for the Cultivation of *Agaricus bisporus*: Relationship between *Clostridia* and Yield. *Microbes Environ ***38**: ME22041.

https://doi.org/10.1264/jsme2.ME22041

## Figures and Tables

**Fig. 1.**
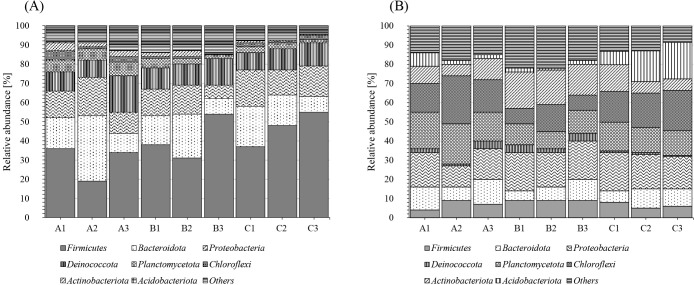
Bacteria present in substrates after primary fermentation (A) and secondary fermentation (B) (phylum level).

**Fig. 2.**
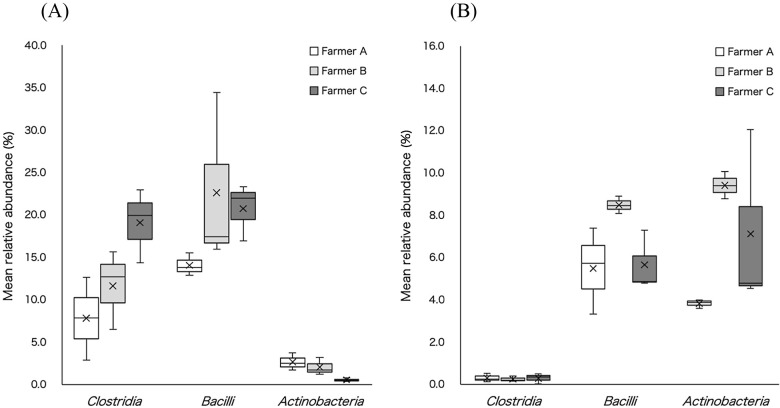
Bacteria present in substrates after primary fermentation (A) and secondary fermentation (B) (class level).

**Fig. 3.**
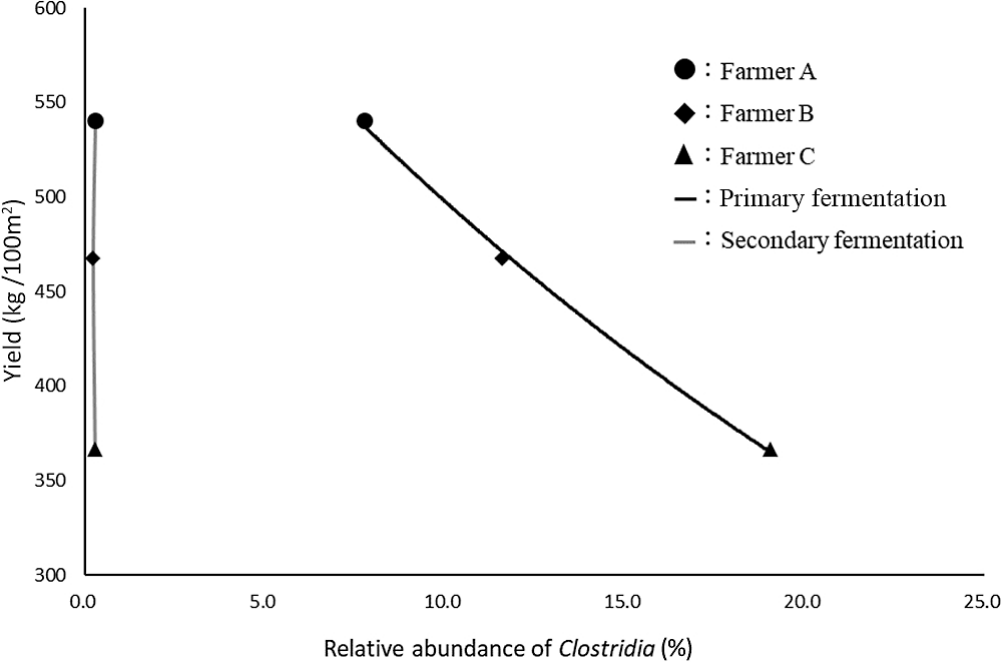
Relationship between *Clostridia* and the yield of *Agaricus bisporus* in post-fermentation substrates. The vertical axis is yield per 100 m^2^ and the horizontal axis is the relative abundance of *Clostridia*. The r-squared value of the graph for primary fermentation was 0.9982. The r-squared value of the graph for secondary fermentation was 0.0086.

**Table 1.** Bacteria present in substrates after primary and secondary fermentation (order level).

		Farmer A (%)	Farmer B (%)	Farmer C (%)
After primary fermentation	*Lactobacillales*	0.382	0.202	0.283
	*Pseudomonadales*	1.929	1.818	3.196
After secondary fermentation	*Lactobacillales*	0.020	0.006	0.000
	*Pseudomonadales*	0.456	0.291	0.693
